# Fruit and vegetable consumption and mortality in Eastern Europe: Longitudinal results from the Health, Alcohol and Psychosocial Factors in Eastern Europe study

**DOI:** 10.1177/2047487315582320

**Published:** 2015-04-22

**Authors:** Denes Stefler, Hynek Pikhart, Ruzena Kubinova, Andrzej Pajak, Urszula Stepaniak, Sofia Malyutina, Galina Simonova, Anne Peasey, Michael G Marmot, Martin Bobak

**Affiliations:** 1 Department of Epidemiology and Public Health, University College London, UK; 2 Centre for Health Monitoring, National Institute of Public Health, Czech Republic; 3 Department of Epidemiology and Population Sciences, Jagiellonian University, Poland; 4 Institute of Internal and Preventive Medicine, Siberian Branch of the Russian Academy of Medical Sciences, Russia; 5 Novosibirsk State Medical University, Russia

**Keywords:** Fruit and vegetable intake, mortality; Central and Eastern Europe, former Soviet Union

## Abstract

**Background:**

It is estimated that disease burden due to low fruit and vegetable consumption is higher in Central and Eastern Europe (CEE) and the former Soviet Union (FSU) than any other parts of the world. However, no large scale studies have investigated the association between fruit and vegetable (F&V) intake and mortality in these regions yet.

**Design:**

The Health, Alcohol and Psychosocial Factors in Eastern Europe (HAPIEE) study is a prospective cohort study with participants recruited from the Czech Republic, Poland and Russia.

**Methods:**

Dietary data was collected using food frequency questionnaire. Mortality data was ascertained through linkage with death registers. Multivariable adjusted hazard ratios were calculated by Cox regression models.

**Results:**

Among 19,333 disease-free participants at baseline, 1314 died over the mean follow-up of 7.1 years. After multivariable adjustment, we found statistically significant inverse association between cohort-specific quartiles of F&V intake and stroke mortality: the highest vs lowest quartile hazard ratio (HR) was 0.52 (95% confidence interval (CI): 0.28–0.98). For total mortality, significant interaction (*p* = 0.008) between F&V intake and smoking was found. The associations were statistically significant in smokers, with HR 0.70 (0.53–0.91, *p* for trend: 0.011) for total mortality, and 0.62 (0.40–0.97, *p* for trend: 0.037) for cardiovascular disease (CVD) mortality. The association was appeared to be mediated by blood pressure, and F&V intake explained a considerable proportion of the mortality differences between the Czech and Russian cohorts.

**Conclusions:**

Our results suggest that increasing F&V intake may reduce CVD mortality in CEE and FSU, particularly among smokers and hypertensive individuals.

## Introduction

A number of observational epidemiological studies have found inverse associations between fruit and vegetable (F&V) intake and the risk of cardiovascular disease (CVD), coronary heart disease (CHD) and stroke.^1–4^ The mechanisms how F&V intake might reduce disease risk are not entirely clear; while the antioxidant hypothesis was not confirmed,^5^ blood pressure lowering effect of F&V consumption, confirmed in experimental trials, seems to be an important mediator.^6^

Although most studies of F&V intake and CVD have been carried out in Western European or North American populations, low F&V consumption has been suggested to be one of the reasons for the high CVD mortality rates in countries of Central and Eastern Europe (CEE) and the former Soviet Union (FSU).^7,8^ The World Health Organisation (WHO) Global Burden of Disease (GBD) project has estimated that the disease burden due to inadequate F&V consumption is higher in CEE/FSU than any other parts of the world.^9^ Despite this indirect evidence, reliable individual-level dietary data in CEE and FSU countries are scarce and, to date, no well-powered studies of F&V intakes in relation to CVD have been reported in the region.

In this study, we investigated the relationship between F&V intake and mortality from all-causes, CVD, CHD and stroke in three populations participating in the Health, Alcohol and Psychosocial Factors in Eastern Europe (HAPIEE) project,^10^ the largest cohort study with population data on dietary habits in CEE and FSU. As secondary objectives, we examined the potential mediating role of blood pressure, and whether F&V intake explained any of the mortality differences between the study populations.

## Methods

### Subjects

Between 2002–2005, 28,945 middle-aged man and women, randomly selected from population/electoral registers in Krakow (Poland), Novosibirsk (Russia) and six cities of the Czech Republic (Havířov/Karviná, Jihlava, Ústí nad Labem, Liberec, Hradec Králové, and Kroměříz), were recruited for the HAPIEE study. The overall response rate was 59%.^10^ Participants completed an extensive questionnaire, provided a blood sample and underwent a medical examination. Written informed consent was provided by all participants, and the study protocols were approved by ethical committees at University College London and all participating centres.

Individuals whose mortality data was not available (*n* = 1048), provided implausible dietary data (participants in the lowest and highest 1% of the energy intake vs basal metabolic rate ratio distribution) (*n* = 548), had not answered more than 10% of the questions on the food frequency questionnaire (FFQ) (n = 685) or had indicated that the FFQ was not representative to their diet (*n* = 806) were excluded from the analysis. Similarly, those with previously diagnosed CVD or diabetes (*n* = 6525) were also excluded. After these exclusions, 19,333 participants (5967 Czech, 6543 Polish and 6823 Russian) were included in the analysis.

### Dietary assessment

Dietary data collection in HAPIEE study has been described in detail elsewhere.^11^ Briefly, a food frequency questionnaire (FFQ) was used to assess the participants’ eating habits in the previous three months. The Czech, Polish and Russian questionnaires consisted of 136, 148 and 147 food and drink items, respectively.

The European Food Safety Authority`s FoodEx 2 food classification and description system was used to categorise food items into fruit and vegetable food groups.^12^ All items which are listed in the group of ‘fresh fruits’ (A04RK) or ‘vegetable and vegetable products’ (A00FJ), with the exception of ‘vegetable products’ (A00ZA), were considered as fruits and vegetables. Overall, 21 fruit and 24 vegetable items were included (Supplementary Material, Table S1).

Participants indicated how frequently they consumed a portion of a particular food item on a nine-point scale. Daily consumption of the different F&V items were calculated by multiplying the number of portions per day by average portion sizes determined by local dieticians. A person’s daily F&V consumption was calculated by adding up the intake values of the different items.

As self-reported dietary intakes are often imprecise, we assessed the validity of F&V intake data against plasma biomarker concentrations, measured in a random sub-sample of participants in all three countries and determined in a central laboratory (Clinical Trials Service Unit, Oxford). In the pooled sample, the cohort, sex and energy intake adjusted Pearson`s partial correlation coefficients between fruit intake and vitamin C and beta-carotene plasma concentrations were 0.29 and 0.05, respectively. The correlation coefficients for vegetable intake were 0.11 and 0.17, respectively.^13^

### Mortality follow-up

Local and regional death registers in Krakow and Novosibirsk and national death register in the Czech Republic were used to identify deaths amongst the participants. Causes of death were determined using the 9th and 10th revision of the International Classification of Diseases (ICD): CVD (ICD-9: 390–459; ICD-10: I00–I99), CHD (410–414; I20–I25), stroke (430–438; I60–I69). Seventy deceased participants with no information on the cause of death were included in the analysis if the outcome was all-cause mortality but excluded for CVD, CHD and stroke.

### Statistical analysis

Cox proportional hazard model was applied to estimate the association with all-cause and cause-specific mortality. F&V intake, categorised into cohort-specific quartiles, was used as the main exposure variable. Additionally, we also calculated the hazard ratios (HRs) of mortality per one unit (100 g/day) increase across six absolute intake categories (<100 g/d, 1–200 g/d, 2–300 g/d, 3–400 g/d, 4–500 g/d, >500 g/d). Proportionality assumption was checked using the Schoenfeld residuals. In model 1, the associations of F&V intake with mortality were adjusted for sex, age and cohorts. In model 2, the associations were further adjusted for education (primary or less, vocational, secondary, university), household amenities score (number of household amenities possessed; 0–5: low, 5–7: moderate, 8–12: high), marital status (married/cohabiting, single/divorced/widowed), alcohol intake (abstainers; light drinkers: <15 g/day for women, <30 g/day for men; moderate to heavy drinkers: ≥15 g/day for women, ≥30 g/day for men), smoking (non-, ex-, current smokers), physical activity (inactive, moderately active, active; based on cross-tabulating the sex specific quartiles of leisure time physical activity expressed in metabolic equivalent of task (MET)-hours/day with occupational activity categories), total energy intake (MJ/day), vitamin supplement intake (no intake, irregular intake – less than three times a week, regular intake – at least three times a week) and diet quality (using the healthy diet indicator (HDI) without the F&V component).^13^ Since the correlation between fruit and vegetable intake was moderate (Spearman`s rho = 0.21), the HRs were further adjusted for each other when their association with mortality outcomes were examined separately.

The preventable proportion (PP) of deaths which could be avoided if participants in the lowest three quartiles would shift their intake one quartile upward was calculated using the same formula as in previous studies.^2^

Because of a significant interaction between F&V intake and smoking for all-cause mortality (*p* = 0.008), we also report results separately by smoking groups. Although we found no significant interaction between F&V intake and cohorts, data were also analysed separately by country cohorts.

In order to assess the mediating effect of blood pressure, the associations were further adjusted for mean arterial blood pressure (MAP) in the subsample of participants who were not taking antihypertensive medications (*n* = 13,966). MAP was calculated from systolic blood pressure (SBP) and diastolic blood pressure (DBP) as follows:^14^

MAP=1/3(SBP)+2/3(DBP)

To estimate how much of the mortality differences between cohorts can be explained by F&V intake, age and sex adjusted HRs of cohort-specific mortality rates were further adjusted for F&V intake, using the Czech cohort as reference group and F&V intake as a continuous variable.

Two sided *p*-values < 0.05 were used to identify statistical significance. All statistical analysis was carried out with the statistical software STATA 13.1 (StataCorp, Texas, USA).

### Imputation of missing data

At least one variable used in the analysis (marital status, smoking habits, education, household amenities score, physical activity, body mass index (BMI), vitamin supplement intake, MAP or serum cholesterol) had missing data for 3122 participants (16% of the analytical sample). We assumed that these data were missing at random because sensitivity analysis showed that although the ‘missingness’ was significantly associated with several covariates in all three country cohorts, many of these associations became non-significant when age, sex, smoking and alcohol intake were adjusted for. Multiple random imputation was carried out using the ‘mi impute chained’ command in STATA. Ten imputed datasets were created, and the following predictor variables were included:^15^ age, sex, alcohol intake, total energy intake, F&V intake, follow-up time and all-cause mortality. The procedure was carried out separately for each cohort.

## Results

The median F&V consumption in the pooled sample was 426.7 g/day (Supplementary Material, Table S2). Compared to the other two country cohorts, Russian participants had substantially lower fruit and total F&V intakes. Over a mean follow up of 7.1 years, 1314 deaths occurred. Total and CVD (including CHD and stroke) mortality rates were considerably higher in the Russian cohort than in the Czech and Polish samples.

Table 1 shows the distribution of participants’ socio-demographic and lifestyle characteristics and CVD risk factors across cohort-specific quartiles of F&V intakes. Being female, higher education and higher household amenities score were positively associated with F&V intake. Those who ate more fruits and vegetables also seem to have better overall diet, and were less likely to be heavy drinkers, smokers, or physically inactive. Among the potential mediators, mean arterial blood pressure declined but BMI increased and serum cholesterol level did not change with increasing F&V consumption, which suggests that blood pressure is a possible but BMI and cholesterol are unlikely mediators between F&V intake and CVD.

**Table 1.** Distribution of sample characteristics across cohort-specific fruit and vegetable intake quartiles.

	Cohort-specific fruit and vegetable intake quartiles			
	Q1	Q2	Q3	Q4
*F&V intake*
Median fruit intake (IQR), g/day	75.2 (36.4–127.1)	170.2 (95.7–246.0)	268.8 (158.0–369.8)	482.3 (306.6–686.7)
Median vegetable intake (IQR), g/day	119.4 (80.3–161.8)	189.4 (138.1–234.1)	247.0 (183.1–318.0)	371.3 (262.6–495.4)
Median fruit and vegetable intake (IQR), g/day	214.1 (165.2–251.3)	352.1 (318.7–412.6)	514.7 (449.1–591.1)	831.4 (698.5–1067.4)
*Socio-demographic characteristics*
Mean age (SD), years	57.1 (7.1)	57.0 (7.1)	57.1 (7.0)	56.7 (6.8)
Sex, female, %	42.7	51.2	58.5	65.8
Marital status,^a^ married, %	72.2	76.6	76.2	76.5
Education,^a^ primary or less, %	11.2	10.0	10.1	8.6
Education,^a^ university, %	23.3	25.0	25.3	29.3
Household amenities score,^a^ low, %	27.4	21.5	19.5	16.7
Household amenities score,^a^ high, %	28.4	32.5	34.8	38.7
*Lifestyle characteristics*
Mean energy intake (SD), MJ/day	8.4 (2.6)	9.2 (2.8)	9.8 (2.9)	11.2 (3.3)
Mean HDI score (without F&V component) (SD)	45.3 (8.8)	45.6 (8.4)	46.4 (8.6)	46.2 (8.4)
Median alcohol intake (IQR), g/day	1.9 (0.2–11.0)	1.7 (0.2–8.5)	1.2 (0.2–6.7)	1.0 (0.1–5.7)
Alcohol, moderate to heavy drinkers, %	12.0	9.7	7.6	7.0
Smoking,^a^ current smokers, %	38.6	30.5	27.7	25.9
Physical activity,^a^ low, %	50.1	49.0	48.3	47.6
Vitamin supplement intake, regular, %	13.3	15.4	19.9	22.9
*Possible mediators*
Mean BMI,^a^ (SD), kg/m^2^	27.3 (4.7)	27.7 (4.7)	28.0 (4.7)	28.1 (4.8)
BMI^a^ >30 kg/m^2^, %	24.6	27.7	29.0	30.7
Mean MAP,^a^ (SD), mm Hg	105.5 (15.4)	105.2 (15.2)	104.7 (15.2)	103.8 (14.9)
Hypertension,^a,b^ %	46.9	47.7	47.1	44.8
Mean serum cholesterol level,^a^ (SD), mmol/l	6.0 (1.2)	6.0 (1.2)	6.0 (1.2)	6.0 (1.2)
Hypercholesterolaemia,^a,c^ %	75.4	76.6	77.1	77.5

BMI: body mass index; F&V: fruit and vegetable; HDI: healthy diet indicator; IQR: interquartile range; MAP: mean arterial blood pressure; SD: standard deviation.

a Including imputed data; ^b^MAP > 110 mm Hg or on antihypertensive medication; ^c^serum cholesterol level > 5.2 mmol/l or on lipid lowering medication.

The associations between F&V intake and the mortality outcomes are presented in Table 2. Although inverse associations were found for all four mortality outcomes, statistically significant lower mortality risk in the highest compared to the lowest F&V intake quartiles was found only for stroke after multiple adjustment. The trends were borderline significant for CVD and stroke, and non-significant for all-cause and CHD mortality. The PP estimates indicated that if F&V intake increased by one quartile, the reduction in mortality would be the greatest for stroke (16.3%). When the effects of fruit and vegetable intakes were analysed separately, the multivariable adjusted results indicated inverse but mostly statistically non-significant associations.

**Table 2.** Results of Cox regression analysis on the pooled sample (*n* = 19,333).

			Cohort-specific quartiles										Per 100 g/day increase^a^	
			Q1		Q2		Q3		Q4					
Cause of death	Deaths/*n*	Model	HR		HR	(95% CI)	HR	(95% CI)	HR	(95% CI)	*p*-value (trend)	PP%	HR	(95% CI)
*Fruit and vegetable intake*														
All-cause	1314/19,333	Model 1	**1.00**	ref.	0.78	(0.68–0.90)	0.77	(0.66–0.89)	0.67	(0.58–0.79)	<0.001	10.1	0.90	(0.87–0.90)
		Model 2	**1.00**	ref.	0.93	(0.80–1.08)	0.97	(0.83–1.13)	0.91	(0.76–1.08)	0.356	2.4	0.98	(0.94–1.02)
CVD	438/19,263	Model 1	**1.00**	ref.	0.66	(0.51–0.84)	0.65	(0.51–0.84)	0.54	(0.41–0.72)	<0.001	16.1	0.87	(0.81–0.93)
		Model 2	**1.00**	ref.	0.80	(0.62–1.03)	0.83	(0.64–1.09)	0.74	(0.54–1.01)	0.060	7.7	0.95	(0.89–1.02)
CHD	226/19,263	Model 1	**1.00**	ref.	0.62	(0.44–0.87)	0.61	(0.43–0.87)	0.60	(0.41–0.87)	0.003	14.3	0.87	(0.80–0.96)
		Model 2	**1.00**	ref.	0.79	(0.55–1.13)	0.85	(0.59–1.25)	0.92	(0.60–1.39)	0.608	2.4	0.99	(0.89–1.09)
Stroke	109/19,263	Model 1	**1.00**	ref.	0.62	(0.37–1.02)	0.69	(0.42–1.13)	0.50	(0.28–0.88)	0.019	17.9	0.88	(0.77–1.00)
		Model 2	**1.00**	ref.	0.67	(0.40–1.12)	0.73	(0.44–1.24)	0.52	(0.28–0.98)	0.056	16.3	0.91	(0.78–1.05)
*Fruit intake*^b^														
All–cause	1314/19,333	Model 1	**1.00**	ref.	0.75	(0.65–0.86)	0.72	(0.62–0.84)	0.68	(0.58–0.79)	<0.001	10.2	0.92	(0.88–0.96)
		Model 2	**1.00**	ref.	0.95	(0.82–1.10)	0.97	(0.83–1.13)	0.99	(0.83–1.18)	0.845	0.3	**1.00**	(0.96–1.04)
CVD	438/19,263	Model 1	**1.00**	ref.	0.77	(0.61–0.97)	0.55	(0.42–0.72)	0.53	(0.40–0.70)	<0.001	16.6	0.84	(0.78–0.91)
		Model 2	**1.00**	ref.	**1.00**	(0.79–1.28)	0.75	(0.56–0.99)	0.78	(0.57–1.07)	0.034	6.2	0.92	(0.84–0.99)
CHD	226/19,263	Model 1	**1.00**	ref.	0.67	(0.48–0.94)	0.51	(0.35–0.74)	0.54	(0.36–0.80)	<0.001	16.9	0.85	(0.76–0.95)
		Model 2	**1.00**	ref.	0.91	(0.65–1.28)	0.73	(0.49–1.08)	0.86	(0.55–1.33)	0.235	4.0	0.95	(0.85–1.07)
Stroke	109/19,263	Model 1	**1.00**	ref.	0.90	(0.57–1.43)	0.62	(0.37–1.05)	0.51	(0.28–0.93)	0.011	16.0	0.82	(0.70–0.97)
		Model 2	**1.00**	ref.	1.12	(0.69–1.82)	0.79	(0.45–1.38)	0.66	(0.34–1.29)	0.164	9.4	0.87	(0.73–1.03)
*Vegetable intake*^b^														
All-cause	1314/19,333	Model 1	**1.00**	ref.	0.76	(0.65–0.88)	0.75	(0.65–0.87)	0.72	(0.62–0.83)	<0.001	8.8	0.93	(0.89–0.97)
		Model 2	**1.00**	ref.	0.82	(0.70–0.95)	0.84	(0.72–0.98)	0.85	(0.72–1.00)	0.052	4.4	0.98	(0.93–1.03)
CVD	438/19,263	Model 1	**1.00**	ref.	0.83	(0.65–1.06)	0.69	(0.53–0.89)	0.67	(0.51–0.88)	<0.001	10.3	0.90	(0.84–0.98)
		Model 2	**1.00**	ref.	0.93	(0.72–1.20)	0.81	(0.62–1.07)	0.88	(0.66–1.19)	0.249	3.2	0.99	(0.90–1.07)
CHD	226/19,263	Model 1	**1.00**	ref.	0.81	(0.58–1.15)	0.65	(0.45–0.94)	0.71	(0.49–1.02)	0.027	9.2	0.91	(0.82–1.01)
		Model 2	**1.00**	ref.	0.94	(0.66–1.34)	0.82	(0.55–1.20)	**1.00**	(0.66–1.51)	0.745	0.0	1.01	(0.89–1.14)
Stroke	109/19,263	Model 1	**1.00**	ref.	0.73	(0.44–1.21)	0.64	(0.38–1.08)	0.64	(0.38–1.07)	0.066	12.1	0.91	(0.78–1.06)
		Model 2	**1.00**	ref.	0.76	(0.45–1.26)	0.65	(0.38–1.13)	0.69	(0.39–1.24)	0.157	10.0	0.94	(0.79–1.12)

CI: confidence interval; CHD: coronary heart disease; CVD: cardiovascular disease; HDI: healthy diet indicator; HR: hazard ratio; PP: preventable proportion.

Model 1: adjusted for sex, age, cohort. Model 2: adjusted for sex, age, cohort, alcohol intake, smoking, education, household amenities score, marital status, energy intake, physical activity, vitamin supplement intake, HDI (without F&V component), fruit/vegetable intake.

a Per one unit increase across six intake categories (<100 g/d, 1–200 g/d, 2–300 g/d, 300–400 g/d, 400–500 g/d, >500 g/d).

b In model 2, fruit and vegetable intakes were mutually adjusted for each other.

In the subgroup analysis, we found statistically significant inverse associations between overall F&V intake and total mortality in current smokers but not in ex- or never smokers (Table 3). Significantly reduced CVD and stroke mortality risk in the highest vs lowest intake quartiles was also found only for smokers. In cohort-specific analysis, similarly to the pooled sample, most associations were found to be inverse but statistically not significant (Supplementary Material, Table S3).

**Table 3.** Results of Cox regression analysis by smoking groups.

			Cohort-specific fruit and vegetable intake quartiles										Per 100 g/day increase^a^	
			Q1		Q2		Q3		Q4					
Cause of death	Subgroup	Deaths/*n*	HR		HR	(95% CI)	HR	(95% CI)	HR	(95% CI)	*p*-value (trend)	PP%	HR	(95% CI)
All-cause	Current smokers	638/5905	1.00	ref.	0.90	(0.73–1.11)	0.87	(0.70–1.09)	0.70	(0.53–0.91)	0.011	8.8	0.93	(0.87–0.98)
	Ex-smokers	300/4080	1.00	ref.	0.94	(0.69–1.28)	0.93	(0.67–1.29)	1.09	(0.76–1.57)	0.748	−2.3	1.00	(0.92–1.10)
	Never smokers	369/9272	1.00	ref.	1.02	(0.76–1.38)	1.22	(0.91–1.66)	1.20	(0.86–1.67)	0.168	−4.5	1.05	(0.97–1.14)
CVD	Current smokers	226/5871	1.00	ref.	0.75	(0.53–1.06)	0.78	(0.54–1.14)	0.62	(0.40–0.97)	0.037	11.9	0.94	(0.85–1.04)
	Ex-smokers	94/4062	1.00	ref.	0.93	(0.55–1.57)	0.71	(0.39–1.32)	1.06	(0.55–2.03)	0.782	−1.6	0.92	(0.79–1.08)
	Never smokers	117/9254	1.00	ref.	0.79	(0.47–1.32)	1.06	(0.64–1.77)	0.80	(0.44–1.45)	0.747	5.5	1.01	(0.87–1.16)
CHD	Current smokers	125/5871	1.00	ref.	0.72	(0.44–1.16)	0.82	(0.50–1.37)	0.76	(0.43–1.35)	0.340	7.3	0.98	(0.86–1.12)
	Ex-smokers	49/4062	1.00	ref.	1.07	(0.52–2.20)	0.52	(0.20–1.37)	1.48	(0.63–3.47)	0.828	−11.9	0.91	(0.73–1.13)
	Never smokers	51/9254	1.00	ref.	0.76	(0.34–1.71)	1.32	(0.62–2.85)	0.97	(0.39–2.40)	0.710	0.8	1.10	(0.88–1.37)
Stroke	Current smokers	50/5871	1.00	ref.	0.76	(0.37–1.56)	0.66	(0.30–1.46)	0.30	(0.10–0.94)	0.038	25.6	0.85	(0.68–1.06)
	Ex-smokers	18/4062	1.00	ref.	0.70	(0.15–3.23)	1.86	(0.49–7.00)	2.09	(0.49–8.87)	0.172	−19.3	1.34	(0.91–1.98)
	Never smokers	41/9254	1.00	ref.	0.55	(0.23–1.30)	0.57	(0.24–1.34)	0.43	(0.16–1.17)	0.110	22.1	0.85	(0.66–1.08)

CI: confidence interval; CHD: coronary heart disease; CVD: cardiovascular disease; HDI: healthy diet indicator; HR: hazard ratio; PP: preventable proportion.

All HRs are adjusted for sex, age, cohort, alcohol intake, education, household amenities score, marital status, energy intake, physical activity, vitamin supplement intake, HDI (without F&V component).

a Per one unit increase across six intake categories (<100 g/d, 100–200 g/d, 200–300 g/d, 300–400 g/d, 400–500 g/d, >500 g/d).

To assess potential mediating role of blood pressure, we conducted analysis with and without additional adjustment for mean arterial blood pressure (MAP) on a subsample of participants who took no antihypertensive medication at baseline (Table 4). After adjusting for MAP, HRs increased for all four mortality outcomes. The reduction in the strength of the association was largest for CVD (the change in the HR between highest vs lowest quartile was 37%).

**Table 4.** Results of Cox regression analysis before and after adjustment for mean arterial blood pressure (MAP) on a subsample of participants who took no antihypertensive medications (*n* = 13,966).

			Cohort-specific fruit and vegetable intake quartiles								Per 100 g/day increase^a^	
			Q1		Q2		Q3		Q4			
Cause of death	Deaths/*n*	Model	HR		HR	(95% CI)	HR	(95% CI)	HR	(95% CI)	HR	(95% CI)
All-cause	939/13, 966	Model 1	1.00	ref.	0.92	(0.77–1.09)	0.94	(0.78–1.13)	0.88	(0.71–1.08)	0.95	(0.91–1.00)
		Model 2	1.00	ref.	0.92	(0.77–1.09)	0.96	(0.80–1.15)	0.90	(0.73–1.11)	0.96	(0.91–1.01)
	Percentage change^b^ (%)				0		33.3		16.7		20.0	
CVD	305/13, 915	Model 1	1.00	ref.	0.79	(0.58–1.07)	0.76	(0.55–1.06)	0.81	(0.56–1.17)	0.94	(0.87–1.03)
		Model 2	1.00	ref.	0.81	(0.60–1.10)	0.80	(0.58–1.12)	0.88	(0.61–1.28)	0.96	(0.88–1.05)
	Percentage change^b^ (%)				9.5		16.7		36.8		33.3	
CHD	175/13, 915	Model 1	1.00	ref.	0.71	(0.47–1.07)	0.76	(0.49–1.18)	0.99	(0.62–1.57)	0.98	(0.87–1.10)
		Model 2	1.00	ref.	0.73	(0.49–1.11)	0.80	(0.51–1.24)	1.07	(0.67–1.71)	1.00	(0.89–1.12)
	Percentage change^b^ (%)				6.9		16.7		na		100.0	
Stroke	65/13 915	Model 1	1.00	ref.	0.81	(0.43–1.53)	0.66	(0.32–1.34)	0.53	(0.23–1.22)	0.86	(0.71–1.05)
		Model 2	1.00	ref.	0.85	(0.45–1.60)	0.72	(0.35–1.48)	0.62	(0.26–1.44)	0.89	(0.73–1.08)
	Percentage change^b^ (%)				21.1		17.6		19.1		21.4	

CI: confidence interval; CHD: coronary heart disease; CVD: cardiovascular disease; HDI: healthy diet indicator; HR: hazard ratio; na: not applicable; PP: preventable proportion.

Model 1: adjusted for sex, age, cohort, alcohol intake, smoking, education, household amenities score, marital status, energy intake, physical activity, vitamin supplement Intake, HDI (without F&V component).

Model 2: adjusted for all covariates in model 1 + MAP.

a Per one unit increase across six intake categories (<100 g/d, 100–200 g/d, 200–300 g/d, 300–400 g/d, 400–500 g/d, >500 g/d)

b % change = (HR2–HR1)/(1–HR1)*100.

When the associations between cohorts and mortality rates were adjusted for F&V intake, the HRs, which reflect the mortality differences between the Czech and the other two cohorts, decreased considerably (Table 5).

**Table 5.** Hazard ratios of cohort differences in all-cause, cardiovascular disease (CVD), coronary heart disease (CHD) and stroke mortality with and without adjustment for fruit and vegetable intake.

Cause of death	Cohort	Model 1	Model 2	Percentage change (%)^a^
		HR (95% CI)	HR (95% CI)	
**All-cause**	**Czech**	1.0	1.0	
	**Polish**	1.22 (1.05–1.42)	1.21 (1.04–1.40)	4.5
	**Russian**	1.91 (1.66–2.19)	1.82 (1.58–2.10)	9.9
**CVD**	**Czech**	1.0	1.0	
	**Polish**	1.14 (0.86–1.51)	1.11 (0.84–1.48)	21.4
	**Russian**	2.87 (2.25–3.68)	2.62 (2.04–3.36)	13.4
**CHD**	**Czech**	1.0	1.0	
	**Polish**	1.23 (0.80–1.90)	1.21 (0.79–1.87)	8.7
	**Russian**	4.13 (2.87–5.96)	3.86 (2.67–5.60)	8.6
**Stroke**	**Czech**	1.0	1.0	
	**Polish**	1.26 (0.66–2.42)	1.22 (0.64–2.35)	15.4
	**Russian**	4.70 (2.74–8.04)	4.15 (2.40–7.16)	14.9

CI: confidence interval; F&V: fruit and vegetable; HR: hazard ratio.

Model 1: adjusted for age, sex.

Model 2: adjusted for age, sex, F&V intake (as continuous variable).

a Percentage change = (HR1–HR2)/(HR1–1)*100.

## Discussion

### Summary of main findings

In this study in three CEE/FSU populations, total and CVD mortality was inversely associated with F&V intake, however, most associations were not statistically significant. We found that the inverse associations were stronger among smokers. The impact of F&V consumption was largest for stroke mortality, and the blood pressure lowering effect of F&V intake appeared as an important mediator. Finally, our results suggest that some of the large mortality gap between the Czech and Russian cohorts might be partially due to the lower F&V intake in Russia.

### Limitations/strengths

Our study has several limitations. The restriction of study participants to urban adults and the moderate response rate restricts the generalizability of our findings, and the exclusions of participants due to missing or implausible data may potentially aggravate the non-response bias. However, the response rate was similar to other recent studies in the CEE/FSU region^16^ and elsewhere, and sensitivity analyses showed that changes in the exclusion/inclusion criteria had little impact on the overall results (data not shown). Although the results may not be entirely applicable to the Czech, Polish and Russian populations as a whole, this limitation does not affect the results regarding the association between F&V intake and mortality outcomes.

A well-known disadvantage of the FFQ is that it tends to over-estimate F&V intake.^17^ If the extent of over-reporting is larger in those with low actual F&V intake, then the variation in reported F&V intake is reduced and effect estimates are under-estimated. This is consistent with the biomarker validation results, which suggested that the misreporting of F&V intake was largest amongst Polish participants,^13^ the cohort in which the association of F&V intake with total and CVD mortality was the weakest. This might help explain the lack of strong associations on the pooled sample.

We cannot exclude the possibility that unmeasured socio-economic or lifestyle factors may have affected (and confounded) the associations. However, the fact that we adjusted for a large number of possible confounders, including the healthy diet indicator (taking into account other dietary factors), reduced the possibility of residual confounding.

On the other hand, the large sample size, the prospective design and the substantial variation of F&V intake both within and between cohorts are important strengths and make this study unique in the research on mortality in CEE/FSU to date.

### Consistency with other studies

The most recent meta-analysis found that the pooled HRs (95% CIs) of all-cause and CVD mortality per one serving/day increase in F&V intake was 0.95 (0.92–0.98) and 0.96 (0.92–0.99), respectively.^18^ This meta-analysis and previous studies indicated similar effects for CHD and stroke and for the associations with fruits and vegetables separately.^3,4,18^ Our results in the pooled HAPIEE sample suggest somewhat weaker link for many intake-outcome pairs, which can be due to the relatively short follow-up time, insufficient statistical power, and potential misclassification of F&V intake.^19^

We found that the inverse association between F&V intake and mortality was significantly stronger in current smokers compared to non-smokers, suggesting that smokers would benefit the most if their F&V consumption was increased. Similar effect of F&V intake in smokers has been described in several,^20,21^ but not all,^22^ previous studies. There are a number of possible explanations for this interaction. For example, as smokers are subject of increased levels of oxidative stress, the protective effect of antioxidants in F&V might be more pronounced for them compared to non-smokers. However, the lack of association between antioxidant vitamins and health outcomes in experimental trials does not support this hypothesis.^23^ F&V contain large amounts of polyphenols as well, and their vasodilator, anti-inflammatory and antithrombotic effects can also counteract the harmful effects of tobacco smoke.^24^ On the other hand, we cannot exclude the possibility of residual confounding, however, when we further adjusted the results for the number of cigarettes smoked per day and the number of years have smoked, the associations remained statistically significant.

The proportion of deaths, which could be prevented if the intake of F&V increased in the population, was higher in our study than in recently published report in Western European populations.^2^ This seems to indicate that the burden of disease, in particular CVD, due to inadequate F&V intake may be higher in Eastern European countries than in Western Europe, consistent with the Burden of Disease calculations.^9^ Further research, using comparable dietary assessment methods in Eastern and Western European samples, would be needed to clarify this question.

The finding that F&V intake was related to decreased blood pressure, which, in turn, contributed to the CVD risk reduction, has been reported in a number of observational and interventional studies.^1,6,25^ This pathway could be involved in the stronger inverse association of F&V intake with stroke compared to CHD. Although the mechanism how F&V intake reduces blood pressure is not entirely clear, the evidence regarding the antihypertensive effects of potassium and magnesium compounds of F&V seems fairly strong,^26^ and some authors also suggest that antioxidants might affect arterial stiffness too.^27^

The low and declining F&V consumption in Russia and other FSU countries was described by Abe and colleagues.^28^ Our study confirmed lower F&V intakes in Russia compared to two Central and Eastern European populations, and the results support the plausible hypothesis that low intakes of F&V might contribute to high CVD mortality in this country. Further studies are needed to explore the specific reasons for the inadequate F&V intake in Russia, and to identify population-based preventative strategies that would be the most effective.^29^

Reducing alcohol consumption and tobacco smoking need to be the focus of any public health intervention campaigns in CEE and FSU which aim to decrease the CVD burden of the population.^30^ The recommendation to increase F&V consumption could complement these main targets well, and could be highly beneficial, especially for smokers and hypertensive individuals.

## Supplementary Material

Supplementary material
