# Supplementary material for: Fruit and vegetable consumption and mortality in Eastern Europe: Longitudinal results from the Health, Alcohol and Psychosocial Factors in Eastern Europe study
Source: Eur J Prev Cardiol. 2015 Apr 22;23(5):493–501. doi: 10.1177/2047487315582320 (PMC4767146; doi:10.1177/2047487315582320)
Supplement: Supplementary material [file Supplementary_file_1.pdf]

Table S1 (Supplementary material). Fruit and vegetable items included in the analysis

FOOD GROUPS	ITEMS
**Fruits**	apple, pear, peach, apricot, plum, cherry, strawberry, raspberry, red currant, black currant, gooseberry, blueberry, orange, mandarin, lemon, grapefruit, kiwi, melon, pineapple, banana, grape
**Vegetables**	broccoli, cauliflower, cabbage, Brussels sprouts, garlic, onion, leek, tomato, cucumber, pepper, aubergine, courgette/marrow, sweet maize, green salad (lettuce), spinach, beetroot, carrot, celeriac, turnip/swedes, parsnip, radish, green beans/runner beans, parsley, mushrooms

Table S2 (Supplementary material). Basic demographics, fruit and vegetable intake and mortality follow-up of the analytical sample

		CZECH	POLISH	RUSSIAN	TOTAL
No. participants		5967	6543	6823	19 333
% females		55.6	52.9	55.2	54.6
Mean age (SD)		57.2 (7.1)	56.5 (6.9)	57.3 (7.0)	57.0 (7.0)
Median fruit intake - g/day (IQR)		293.4 (165.4-476.8)	244.8 (139.8-385.6)	103.0 (50.4-188.1)	194.6 (96.8-353.6)
Median vegetable intake - g/day (IQR)		172.7 (106.5-276.3)	196.3 (129.7-297.8)	232.6 (176.4-328.1)	206.0 (135.4-304.1)
Median fruit and vegetable intake - g/day (IQR)		486.8 (308.8-738.1)	463.7 (314.7-657.5)	354.6 (254.7-505.2)	426.7 (285.2-628.4)
Median follow up time - years (IQR)		8.2 (7.8-8.9)	7.1 (6.9-7.7)	6.5 (5.9-7.1)	7.1 (6.7-7.8)
No. deaths (per 1000 person-years):	All-cause	364 (7.5)	388 (8.4)	562 (13.1)	1314 (9.6)
	CVD	106 (2.2)	99 (2.1)	233 (5.4)	438 (3.2)
	CHD	43 (0.9)	45 (1.0)	138 (3.2)	226 (1.6)
	Stroke	18 (0.4)	20 (0.4)	71 (1.7)	109 (0.8)

SD, Standard deviation; IQR, Interquartile Range; CVD, Cardiovascular disease; CHD, Coronary heart disease

Table S3 (Supplementary material). Results of Cox-regression analysis by country cohorts

			Cohort-specific fruit and vegetable intake quartiles										Per 100g/day increase[a]	
			Q1		Q2		Q3		Q4					
Cause of death	Subgroup	Deaths/n	HR		HR	(95%CI)	HR	(95%CI)	HR	(95%CI)	p-value (trend)	PP%	HR	(95%CI)
All-cause	Czech	364/5967	1.00	ref.	0.82	(0.62-1.10)	0.92	(0.68-1.24)	0.94	(0.67-1.32)	0.817	1.6	0.97	(0.90-1.05)
	Polish	388/6543	1.00	ref.	1.00	(0.76-1.31)	1.04	(0.78-1.39)	1.05	(0.75-1.47)	0.716	-1.3	1.01	(0.94-1.09)
	Russian	562/6823	1.00	ref.	0.98	(0.79-1.23)	0.98	(0.77-1.24)	0.85	(0.65-1.12)	0.314	3.9	0.97	(0.91-1.03)
CVD	Czech	106/5965	1.00	ref.	0.59	(0.35-1.00)	0.64	(0.37-1.11)	0.69	(0.37-1.30)	0.197	10.5	0.90	(0.78-1.03)
	Polish	99/6517	1.00	ref.	1.01	(0.59-1.73)	1.29	(0.75-2.21)	0.91	(0.45-1.85)	0.815	2.1	1.06	(0.91-1.24)
	Russian	233/6781	1.00	ref.	0.84	(0.59-1.18)	0.80	(0.55-1.16)	0.77	(0.51-1.16)	0.168	6.8	0.95	(0.86-1.05)
CHD	Czech	43/5965	1.00	ref.	0.51	(0.21-1.22)	0.66	(0.28-1.56)	0.76	(0.29-2.00)	0.564	8.2	0.92	(0.74-1.14)
	Polish	45/6517	1.00	ref.	0.85	(0.37-1.96)	1.45	(0.66-3.15)	0.82	(0.27-2.50)	0.798	4.4	1.11	(0.88-1.38)
	Russian	138/6781	1.00	ref.	0.90	(0.57-1.40)	0.79	(0.47-1.31)	1.07	(0.64-1.80)	0.938	-1.9	1.00	(0.87-1.14)
Stroke	Czech	18/5965	1.00	ref.	0.23	(0.05-1.16)	0.54	(0.14-2.05)	0.59	(0.13-2.68)	0.554	17.2	0.97	(0.68-1.39)
	Polish	20/6517	1.00	ref.	0.91	(0.30-2.81)	0.62	(0.17-2.24)	0.44	(0.09-2.06)	0.250	18.9	0.89	(0.64-1.25)
	Russian	71/6781	1.00	ref.	0.72	(0.38-1.35)	0.84	(0.44-1.59)	0.52	(0.23-1.14)	0.157	15.8	0.89	(0.74-1.08)

All HRs are adjusted for sex, age, alcohol intake, smoking, education, household amenities score, marital status, energy intake, physical activity, vitamin supplement intake, HDI (without F&V component)

[a] per one unit increase across six intake categories (<100g/d, 1-200g/d, 2-300g/d, 3-400g/d, 4-500g/d, >500g/d)

Hazard ratio; CI, Confidence interval; CVD, Cardiovascular disease; CHD, Coronary heart disease; PP, Preventable proportion; HDI, Healthy diet indicator
